# *LOC134466* methylation promotes oncogenesis of endometrial carcinoma through LOC134466/hsa-miR-196a-5p/TAC1 axis

**DOI:** 10.18632/aging.101644

**Published:** 2018-11-28

**Authors:** Hai Xu, Yuan Sun, Zhen Ma, Xin Xu, Lili Qin, Baoping Luo

**Affiliations:** 1 Department of Obstetrics and Gynecology, Huangjiahu Hospital of Hubei University of Chinese Medicine, Wuhan 430065, Hubei, China; 2 College of Pharmacy, Hubei University of Chinese Medicine, Wuhan 430065, Hubei, China; 3 Department of Dermatology, Hubei University of Chinese Medicine, Wuhan 430065, Hubei, China; 4 Department of Obstetrics and Gynecology, Hubei Provincial Hospital of TCM, Wuhan 430065, Hubei, China; 5 Department of Oncology, The First Clinic College of Hubei University of Chinese Medicine, Wuhan 430065, Hubei, China; 6 Department of Oncology, Hubei Provincial Hospital of Traditional Chinese Medicine, Wuhan 430065, Hubei, China

**Keywords:** endometrial carcinoma, DNA methylation, *LOC134466*, hsa-miR-196a-5p, *TAC1*, 5-Aza-2-Deoxycytidine, neuroactive ligand-receptor interaction

## Abstract

To investigate possible mechanism of abnormal methylation of long non-coding RNA (lncRNA) on endometrial carcinoma (EC) progression, we detected the genome methylation profiling of endometrial carcinoma by bioinformatic analysis. Accordingly, gene *LOC134466* was chosen for the further research. We also found that *TAC1* was the target gene of *LOC134466* and miRNA, hsa-miR-196a-5p, might form a connection between *LOC134466* and *TAC1*. The relationship was further proved by dual-luciferase reporter assay. *In vitro* studies, DNA methylation and expression were determined by MSP and qRT-PCR respectively. Cell proliferation, apoptosis and cell cycle were demonstrated by colony formation assay, Annexin V/PI double staining and flow cytometry. Besides, the function of *LOC134466* and *TAC1* in EC was further confirmed by Tumor Xenograft. Our results indicated that EC progression was promoted by hypermethylated *LOC134466* and *TAC1*. Moreover, *TAC1* transcription was regulated by *LOC134466* via hsa-miR-196a-5p binding. *LOC134466* and *TAC1* demethylation by 5-Aza-2-Deoxycytidine inhibited EC cells proliferation and accelerated cell apoptosis. Furthermore, the expression of TACR1, TACR2 and TACR3 was remarkably decreased through *LOC134466* and *TAC1* treatments. Our findings establish a novel regulatory axis, *LOC134466*/hsa-miR-196a-5p/*TAC1.* Downregulation of the axis promoted EC development through TACR3, which further activated neuroactive ligand-receptor interaction.

## Introduction

Endometrial carcinoma (EC) originates in the endometrium [1] and is one of the commonest malignancy of the female genital tract, which ranks fourth in whole malignancies among women [2]. Each year, about 142000 women in the worldwide suffer from endometrial cancer and 42000 women die from this cancer [2]. Though there are some advances in surgery and chemoradiotherapy, many patients have recurrence and distant metastases after these treatments [3]. Key events of mutation in EC have been characterized, but the underlying molecular mechanisms involved in carcinogenesis or identities of tumor suppressor factors are still unclear [4]. Therefore, an understanding of the molecular mechanisms touched upon the pathogenesis of EC must be increased to identify new therapeutic targets and to develop more effective EC treatment strategies.

In cancer research, long non-coding RNAs (lncRNAs) and micro-RNAs (miRNAs) are the most popular noncoding RNAs, and they represent a vital component of tumor biology [5]. lncRNAs are a new class of non-protein coding molecules that are longer than 200 nucleotides. They do not have protein-coding function or encode open reading frames of insufficient lengths [6]. It has been appreciated that lncRNAs contribute to the occurrence and progression of many types of tumours [7,8]. For example, *CCAT1* is a lncRNA that was upregulated in lung squamous cell carcinoma (LSCC) and this upregulation contributes to enhanced cell invasion and migration [9]. In EC, lncRNAs including HOTAIR, H19 and SRA were found to be upregulated [10]. On the other hand, miRNAs, small non-coding RNAs of about 22 bp can induce the interference of RNA with complementary mRNA and play a role in silencing mRNA [11]. Thus, miRNAs are considered to be closely associated with the regulation of gene expression, epigenetic dysfunction and cancer initiation [11]. For example, miR-198 can inhibit lung cancer cell proliferation and induce cell apoptosis by targeting *FGFR1* [12,13]. Furthermore, many studies over the past ten years have begun to reveal the interaction between mammalian lncRNA and miRNA [14], suggesting that non-coding RNAs may form an lncRNA-miRNA-mRNA interaction network to regulate cancer [15,16]. For instance, lncRNA, H19 preferentially bind to let-7 miRNAs, and the interaction between them is important for maintaining breast cancer stem cell [17]. LncRNA-miRNA interaction also affect EC progression. For example, Guo et al. found that lncRNA-GAS5 promoted PTEN expression by inhibiting miR-103. In the current study, we aim to reveal a novel lncRNA-miRNA interaction in EC.

As a typical epigenetic modification, DNA methylation plays critical roles in the occurrence of cancers [18]. For example, expression of many tumor suppressor genes such as *PTEN*, *MMR* were suppresed by DNA hypermethylation and thus cancer progression is promoted [19]. Previous studies revealed that LncRNAs could affect DNA methylation, but also be regulated by histone modification [20]. In colon cancer, for example, DNA methylation was regulated by DNMT1-assocaited lncRNAs, and this affected expression of DACOR1, which activated tumor-supresor pathways in normal cells [21]. In EC, however, limited information can be found regarding association between lncRNA and DNA methylation.

In the present study, we aim to investigate the roles of lncRNA in EC. Specifically, DNA methylation state will be assessed, trying to answer the question whether interplay between lncRNA and DNA methylation contributes to EC progression.

## RESULTS

### Significant differences existed between EC tissues and paired normal tissues

**Figure 1:**
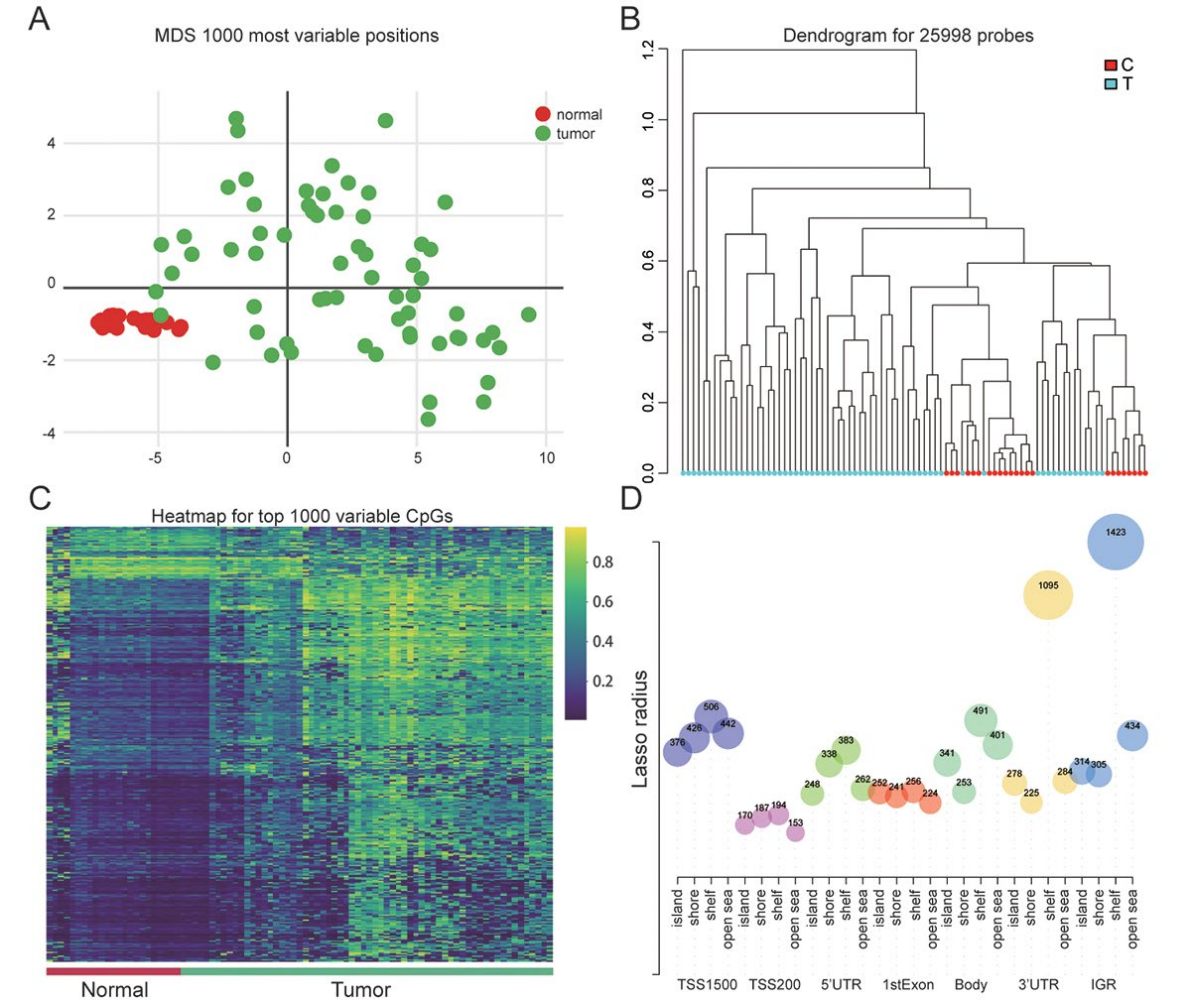
**Genome-wide methylation data for endometrial carcinoma.** (**A**) Multi-dimensional scaling (MDS) plot showing differential clustering of normal vs. tumor samples. (**B**) Dendrogram produced for 25998 probes in normal and tumor samples. (**C**) Heatmap of top 1000 differentially methylated imprinted CpG sites. (**D**) The distribution of CpG sites in different gene regions.

In order to identify 1000 most variable positions, the ways of multi-dimensional scaling were used in which differential clustering between EC and normal were shown (Figure 1A). The dendrogram of 25998 probes visually showed a clear differentiation between normal and endometrial neoplasm (Figure 1B). The heatmap of 1000 variable CpGs showed high methylation in tumor tissues while low methylation in adjacent normal endometrial tissues (Figure 1C). Figure 1D in which the CpGs number in different regions was showed illustrated the CpG content in shelf was the most significant compared with other regions (island,shore, open sea) and IGR contained the largest number of CpGs contrasted with other gene regions (TSS1500, TSS200, 5 'UTR, 1stExon, Body and 3'UTR).

### Different methylation probes’ distribution in gene fragments

**Figure 2:**
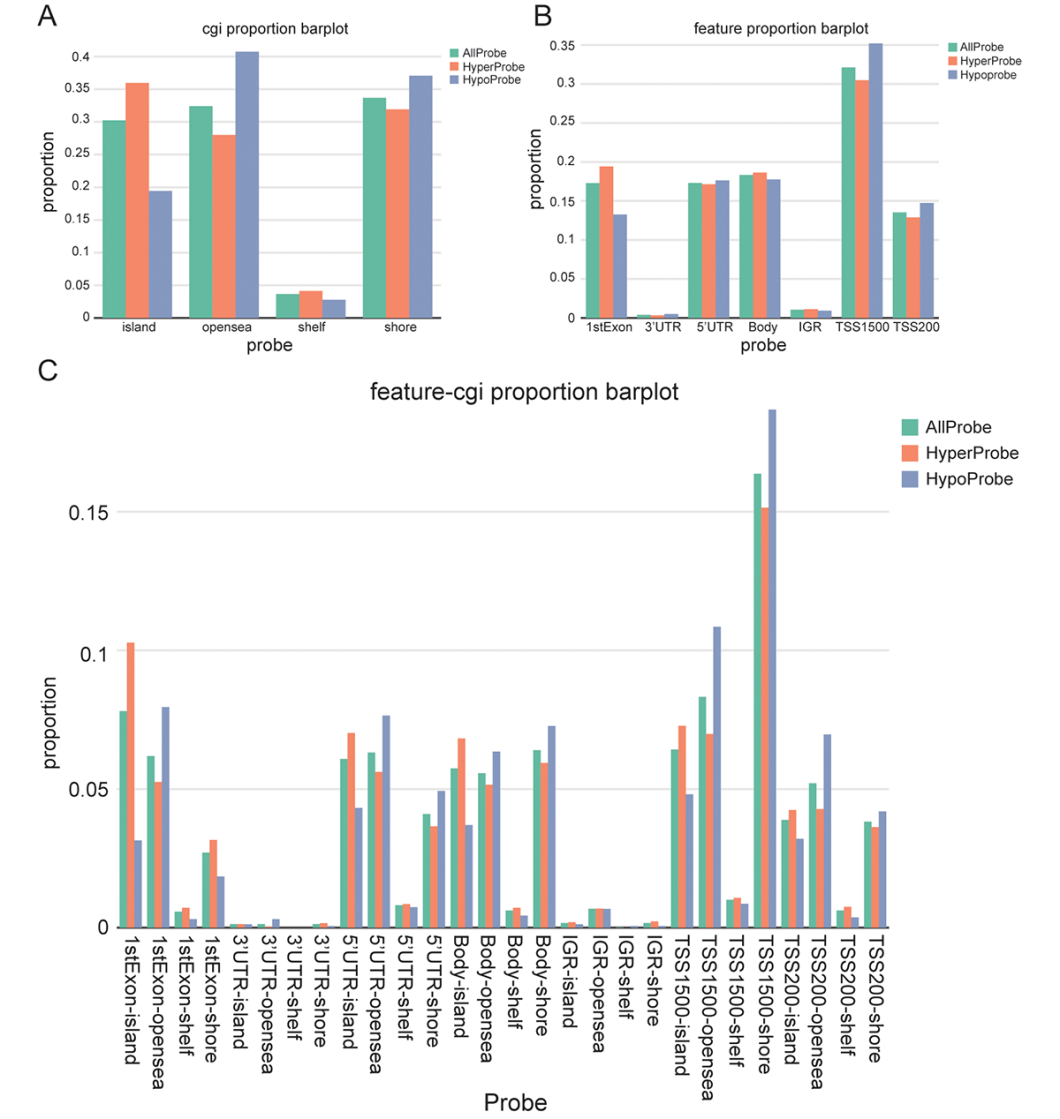
**Screening and visualization of differential CpG sites.** (**A**) Distribution of differentially methylated CpG sites according to regions (shores, shelves, islands, and open sea). (**B**) Distribution of differentially methylated CpG sites according to gene position (1stExon, 3’ UTRs or 5’ UTRs, body, IGR, TSS1500 and TSS200). (**C**) The distribution of differentially methylated CpG sites by combining CpG regions and gene location.

The allocation of different kind of probes at CpG sites externally proved that hyperprobe proportions mainly locates in CpG island, open sea and shore, but not in shelf (Figure 2A). The distribution of probes in different gene fragments regions showed a comprehensive overview about the methylation of gene, and the promoter region’s methylation level inclusive of 5’UTR, TSS1500, TSS200 has reached around 60% (Figure 2B). Combining Figure 2A and Figure 2B, it was found that 1 stExon, 5 ’UTR, body and TSS200 had higher hyperprobe proportions in island, while IGR and TSS1500 had higher hyperprobe proportions in the opensea and shore respectively (Figure 2C).

### LncRNAs and *TAC1* were hypermethylated in EC

**Figure 3:**
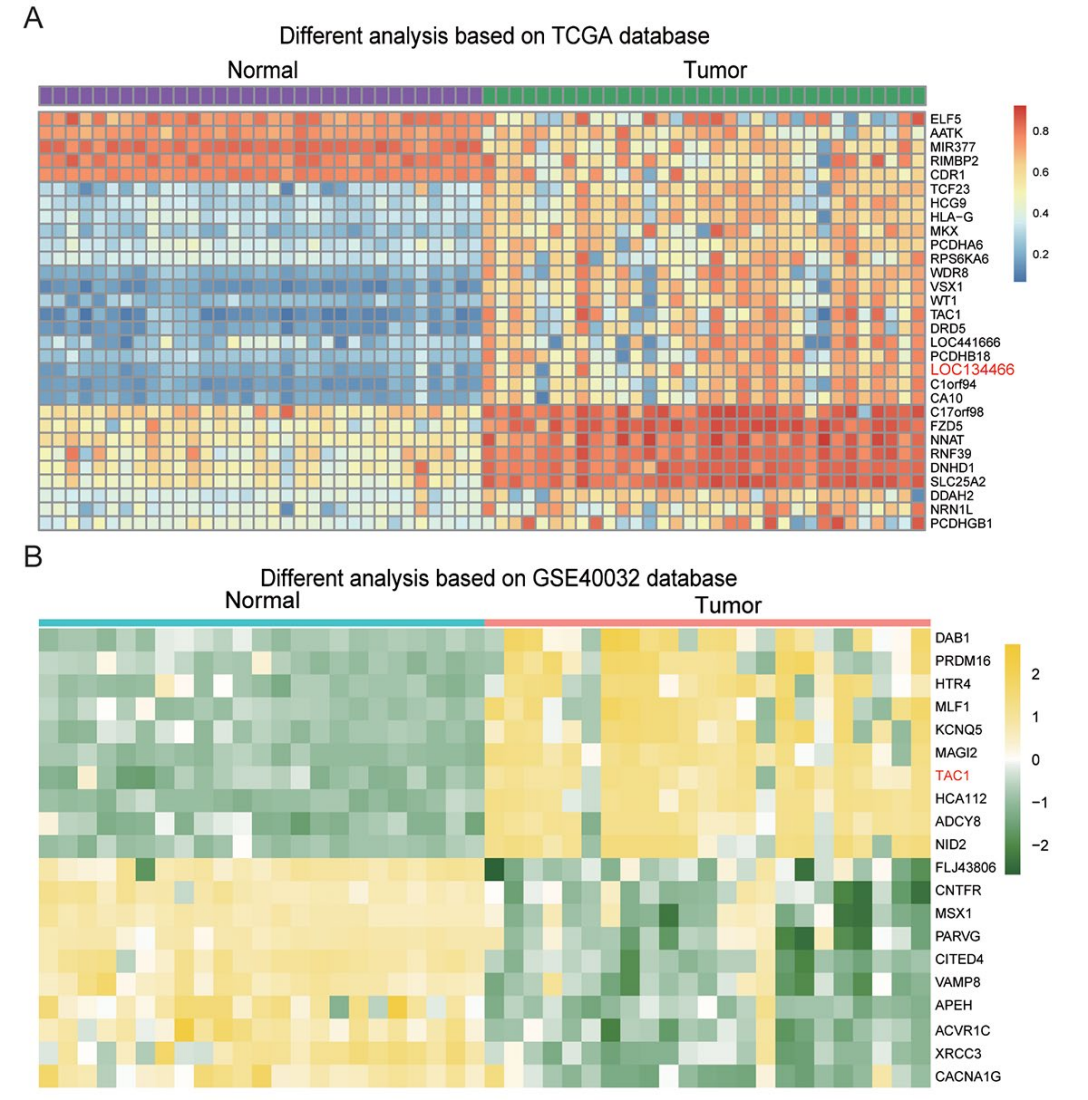
**Hypermethylated genes in endometrial carcinoma.** (**A**) The heatmap showed differentially methylated genes in the 20 endometrial carcinoma samples compared to normal adjacent tissues. (**B**) The heatmap showed differentially methylated genes in endometrial carcinoma compared to paired normal tissues. The results were generated based on GSE40032 database.

Figure 3A showed the 30 most significant differential methylated genes in EC. Two lncRNAs, *LOC 441666* and *LOC134466*, were found to be hypermethylated in EC tissues compared to paired normal tissues. Previous studies have revealed roles of LOC134466 in several cancers, including epithelial ovarian cancer and small cell lung cancer [9,21,22]. Thus, we chose LOC134466 for further studies. On the other hand, 20 differently methylated genes were screened out based on GSE40032 database (Figure 3B). TAC1 gene was the only gene that was identified as hypermethylated gene from both heatmap. Thus, we raised particular interest in TAC1 gene, wondering how lncRNA regulated TAC1 expression if there was a relationship between them.

### CpG analysis of *LOC134466* methylation status

**Figure 4:**
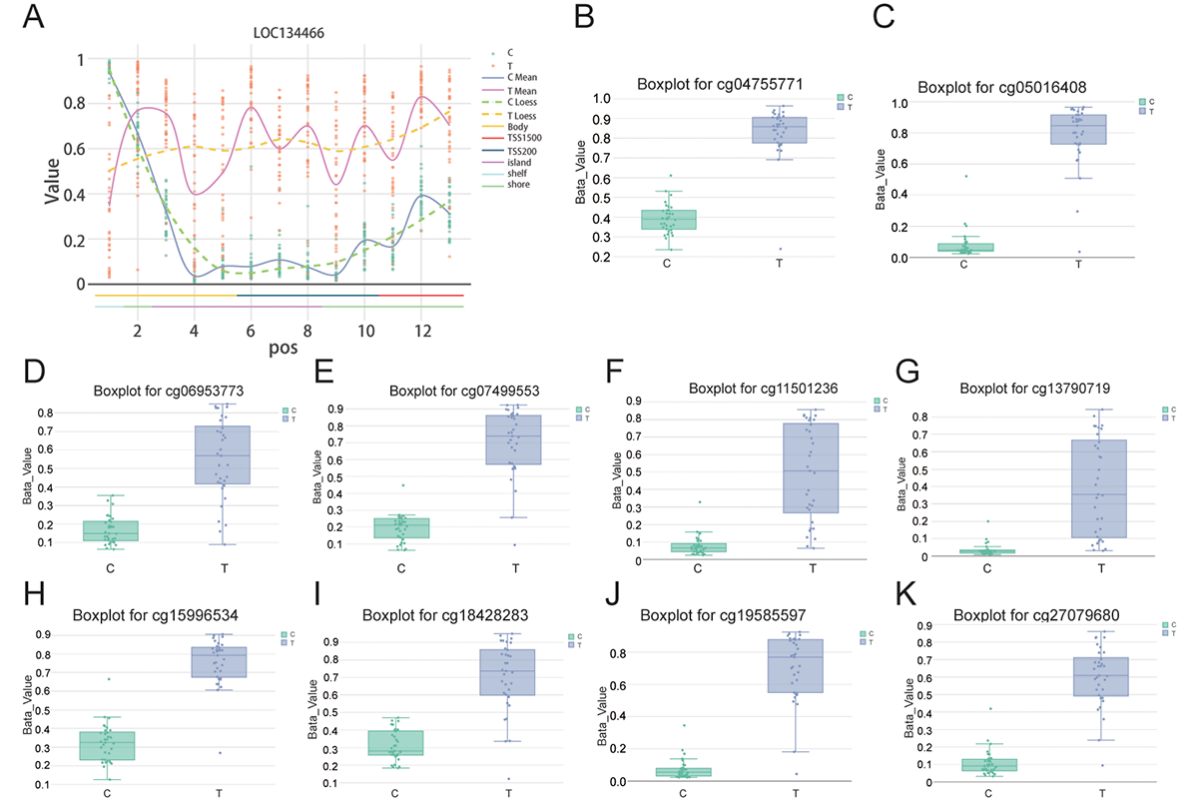
**CpG analysis further proved that *LOC134466* was hypermethylated in EC.** (**A**) *LOC134466* was differentially methylated in tumor tissues compared with paired normal tissues. Boxplot for cg04755771 (**B**), cg05016408 (**C**), cg06953773 (**D**), cg07499553 (**E**), cg11501236 (**F**), cg3790719 (**G**), cg15996534 (**H**), cg18428283 (**I**), cg19585597 (**J**), cg27079680 (**K**), all above showed significant difference in methylation among normal and tumor patients. Beta value was generated by DMP analysis.

As is shown in Figure 4A, *LOC134466* methylation in tumor tissues was higher compared to that in paired normal tissues. From the data of boxplot related to 10 CpG sites that were differentially methylated, we found all CpG sites in the tumor group displayed an increased methylation compared with normal tissues. Boxplot for cg04755771, cg05016408, cg06953773, cg07499553, cg11501236, cg3790719, cg15996534, cg18428283, cg19585597 and cg27079680 were showed in Figure 4B-4K which validated that there was a significant methylation in the tumor group compared with the normal group. To conclude, these results suggested that DNA methylation level for *LOC134466* was increased obviously in endometrial carcinoma tissues.

### Neuroactive ligand-receptor interaction was remarkably up-regulated

**Figure 5:**
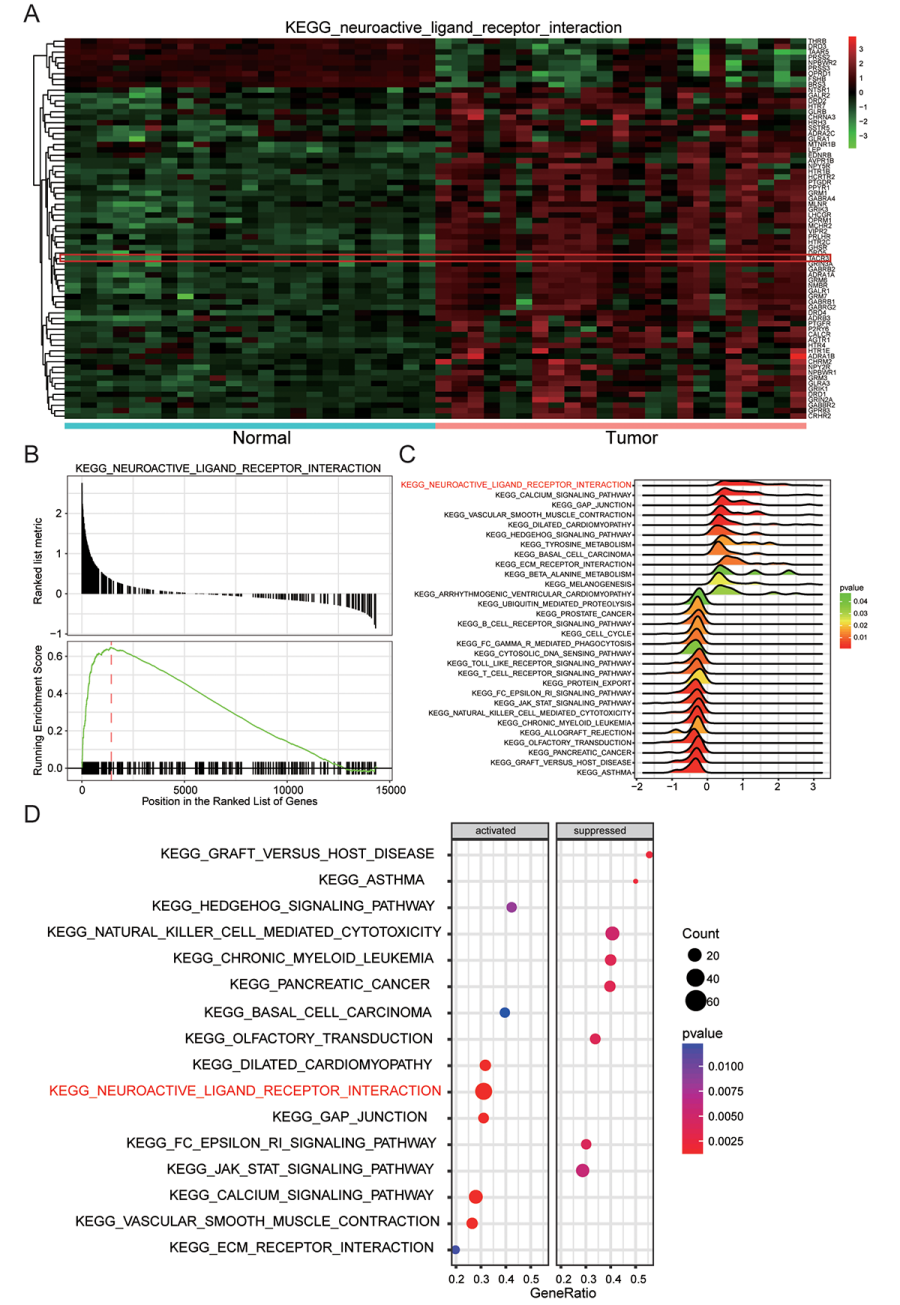
**Neuroactive ligand-receptor interaction was remarkably up-regulated.** (**A**) Genes involving neuroactive ligand-receptor interaction, for example *TACR3*, were generally upregulated. (**B**) Neuroactive ligand-receptor interaction enriched by KEGG was up-regulated. (**C**) Joyplot of GSEA showed that neuroactive ligand-receptor interaction was upregulated in EC. (**D**) Dotplot of GSEA which displayed the activation and inactivation of top 16 significantly enriched signaling pathways showed that neuroactive ligand-receptor interaction was activated in EC.

The heatmap of neuroactive ligand-receptor interaction which closely related with endometrial carcinoma showed hypermethylation of associated genes, including *TACR3*, in tumor tissues and opposite result in normal tissues (Figure 5A). Then on the grounds of GSEA report which was shown in Figure 5B, neuroactive ligand-receptor interaction was discovered to be significantly up-regulated in endometrial carcinoma. And the results of visualized pathway enrichment which was conducted by R and shown as joyplot (Figure 5C) and dotplot (Figure 5D) also revealed the same results.

### Hsa-miR-196a-5p formed a bridge connecting *LOC134466* and *TAC1*

**Figure 6:**
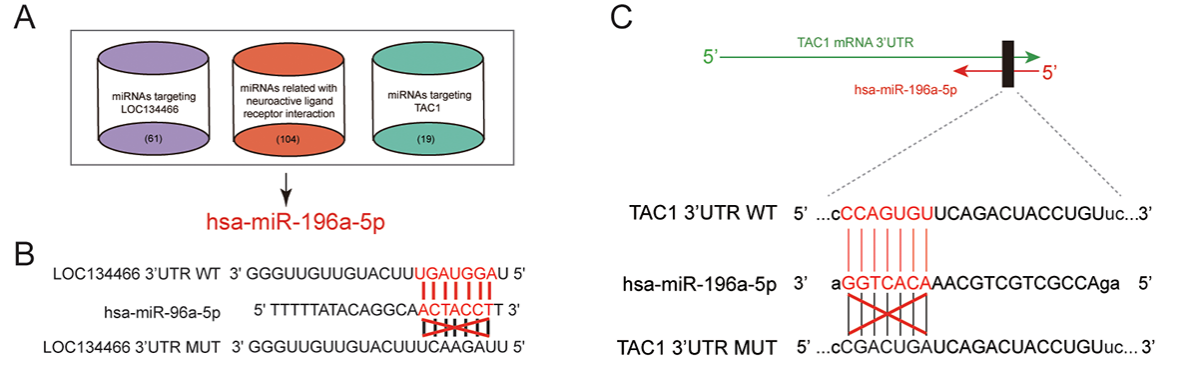
**Analysis of lncRNA/miRNA/mRNA axis.** (**A**) Venn diagram indicated that hsa-miR-196a-5p was a bridge between *LOC134466* and *TAC1*. TargetScan and miRcode database were used to sort out miRNAs that interact with both LOC134466 and TAC1. DIANA Tools was used to screen miRNAs that associate with neuroactive ligand-receptor interaction. (**B**) Predicted binding sites between hsa-miR-196a-5p and *LOC134466*. (**C**) Predicted binding sites between hsa-miR-196a-5p and *TAC1*.

Based on TargetScan as well as miRcode database, we sorted out miRNAs that regulated by *LOC134466*. We also used DIANA Tools to find neuroactive ligand-receptor interaction related miRNAs. As shown in Figure 6A, hsa-miR-196a-5p formed a bridge between *LOC134466* and *TAC1*. Interestingly, hsa-miR-196a-50 was the only miRNA that bound to both *LOC134466* and *TAC1*. Predicted binding sites of *LOC134466* and *TAC1* on hsa-miR-196a-5p gene were shown in Figure 6B-6C. The following experiments aimed to investigate roles of *LOC134466*/hsa-miR-196a-5p/*TAC1* axis on EC progression.

### *LOC134466* and *TAC1* were hypermethylated and downregulated in EC

**Figure 7:**
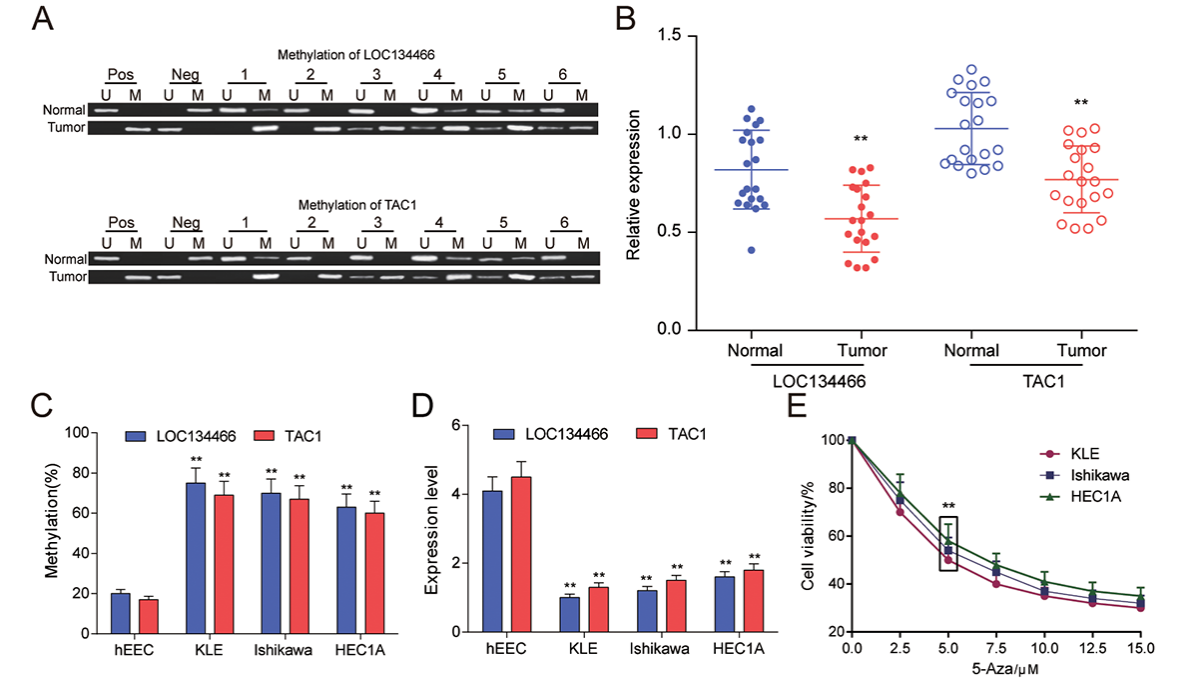
***LOC134466* and *TAC1* were hypermethylated and lower-expressed in EC tumors_._** (**A**) Methylation state of *LOC134466* and *TAC1* in tumor tissues and paired adjacent tissues. There were 20 paired tissue samples and six representative MSP results were represented. (**B**) The expressions of *LOC134466* and *TAC1* in tumor tissues and paired adjacent tissues were assessed by qRT-PCR. The expression of gene was normalized to that of *GADPH*. (**C**) DNA methylation level of *LOC134466* and *TAC1* in normal endometrial cell line (hEEC) and EC tumour cell lines (KLE, Ishikawa and HEC1A) were determined by MSP. (**D**) The *LOC134466* and *TAC1* mRNA expressions in cancer cell lines and normal cell line were analyzed by real-time PCR. The expressions of genes were normalized to that of *GADPH*. (**E**) Minimum effective dose of 5'-Aza-deoxycytidine was determined by cell viability assay. 5 μM 5’-Aza-deoxycytidine showed a great difference. ** *P*<0.01 compared to corresponding control (paired normal tissues or hEEC cell line or cell viability without 5’-Aza treatment).

The results of MSP were shown in Figure 7A. The more distinct white stripe emerged in tumor group indicated that both *LOC134466* and *TAC1* were hypermethylated in EC. Then the expression of the two genes was examined by qRT-PCR. It was found that both *LOC134466* and *TAC1* were dramatically silenced in tumor tissues (Figure 7B), indicating that methylation of gene might negatively affect gene expression. Then, same experiments were conducted using normal endometrial cell line (hEEC) and EC cell lines (KLE, Ishikawa, HEC1A). Consistently, *LOC134466* and *TAC1* were hypermethylated and downregulated in all tumor cell lines (Figure 4C-D). 5-Aza-2-Deoxycytidine concentration screening was done to select the appropriate concentration. The results showed that 5-Aza started to show effects at dose of 5 μM (Figure 7E). To minimize toxicity effects, 5 μM of 5-Aza was used for following studies.

### *LOC134466* and *TAC1* restoration inhibited proliferation and induced apoptosis in EC cells

**Figure 8:**
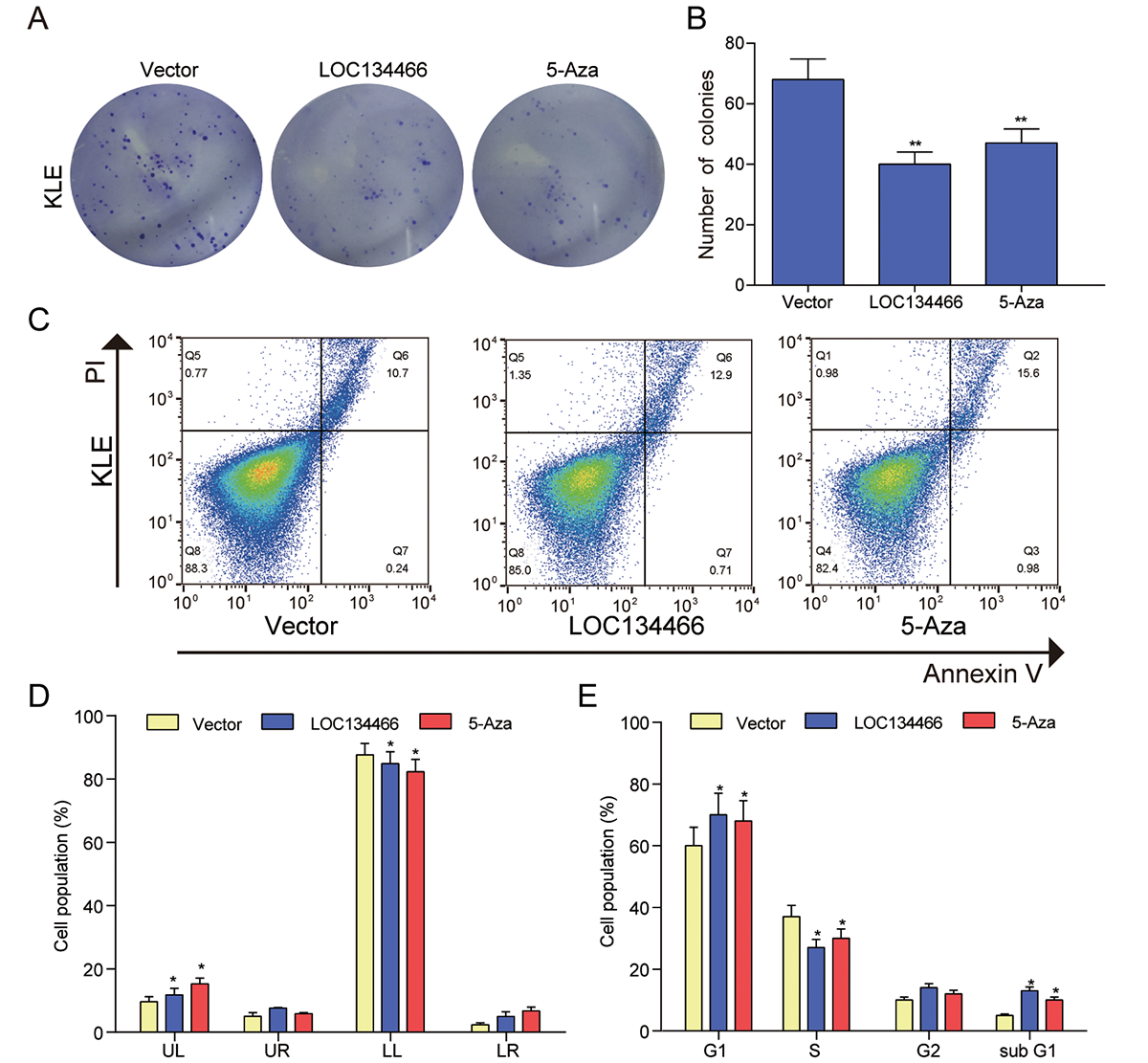
***LOC134466* demethylation inhibited KLE cells proliferation, arrested the cell cycle at the G1 phase, and accelerated cell apoptosis.** (**A-B**) Cell proliferation was assessed by Colony formation assay and proliferation was indicated by number of colonies. (**C-D**) Cell apoptosis was determined by Annexin V/PI double staining and percentages of cells in each phase (LL, viable; LR, early apoptotic; UL and UR, late apoptotic/necrotic cell) were calculated. (**E**) Relative cell numbers in each cell-cycle phase (G1, S, G2, and subG1) were determined by flow cytometer. *P<0.05, **P<0.01, compared with corresponding control group.

**Figure 9:**
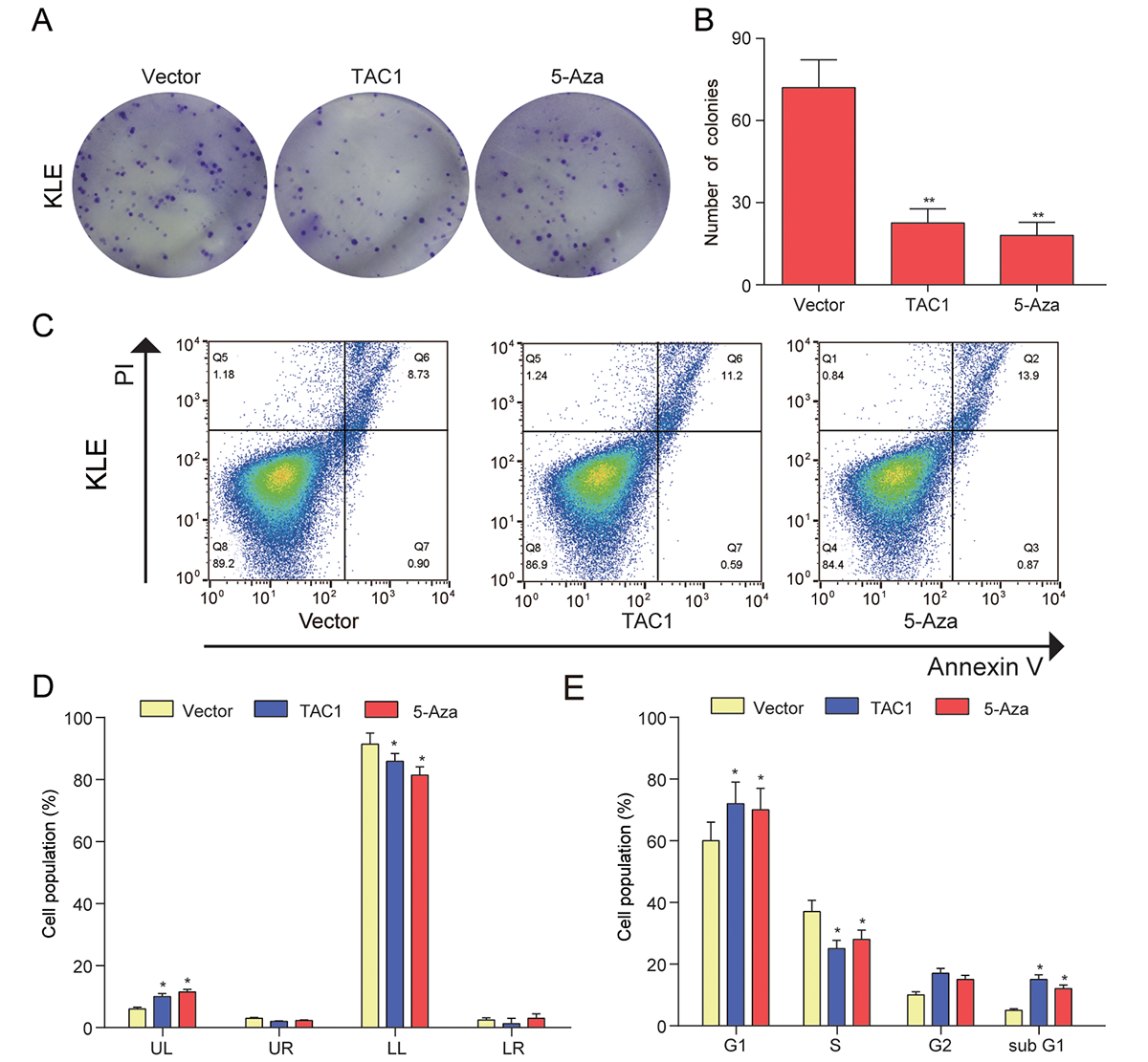
***TAC1* demethylation inhibited KLE cells proliferation, arrested the cell cycle at the G1, and accelerated cell apoptosis.** (**A-B**) Colony formation assay was used to assess cell proliferation ability and proliferation was indicated by number of colonies. (**C-D**) Cell apoptosis was determined by Annexin V/PI double staining and percentages of cells in each phase (LL, viable; LR, early apoptotic; UL and UR, late apoptotic/necrotic cell) were calculated. (**E**) Relative cell numbers in each cell-cycle phase (G1, S, G2, and subG1) were determined by flow cytometer of the *TAC1* and vector control. *P<0.05, **P<0.01, compared with corresponding control group.

The decrease of *LOC134466* and *TAC1* expression in endometrium cancer suggested that the two gens may play a tumor-suppressive role in EC tumorigenesis. To test this notion, we first performed gain-of-function study by overexpressing *LOC134466* or *TAC1* through DNA vector. Successful restoration of *LOC134466* expression (Figure 8A) and *TAC1* expression (Figure 9A) were found to inhibit endometrium carcinoma cell proliferation (Figure 8B&9B). Thereafter, we determine how cell proliferation was modulated by *LOC134466* and *TAC1*. Annexin V/PI double staining revealed that overexpression of *LOC134466* (Figure 8C-9D) and *TAC1* (Figure 9C-9D) induced a reduction in the living cell population (LL phase) and an accompanying increase in the early apoptotic population (LR phase). Moreover, flow cytometer was used to reveal relative cell numbers in each cell-cycle phase (G1, S, G2, and subG1). SubG1 phase refers to some of the debris peaks that existed before the G0 /G1 phase and cells in this phase may be considered as apoptotic cells. A marked increase cells in subG1 was detected when *LOC134466* and *TAC1* were overexpressed (Figure 8E&9E). Noticeably, 5-Aza treatment produced similar effects on cell apoptosis and proliferation as produced by overexpressing *LOC134466* and *TAC1*. The effects of 5-Aza on gene expression were presented in a supplementary figure (Figure S1). As shown in the figure, demethylation restored normal expression of *LOC134466* and *TAC1*. Taken all together, these results suggested that *LOC134466* and *TAC1* overexpression contributed to EC progression.

### Upregulation of *LOC134466*/hsa-miR-196a-5p/TAC1 axis promoted cell proliferation and inhibited apoptosis

**Figure 10:**
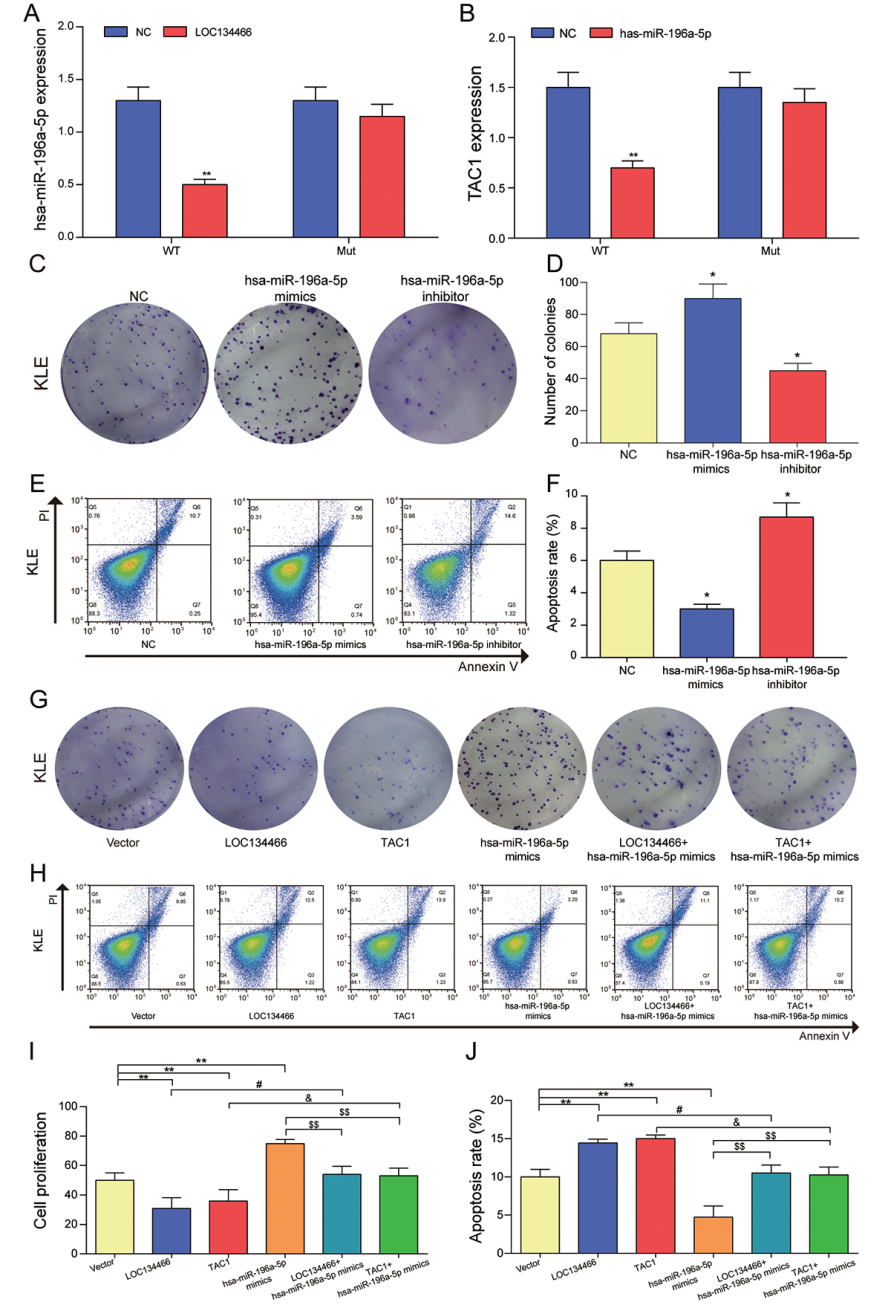
**The relationship among *LOC134466*, hsa-miR-196a-5p and *TAC1*, and the effects of hsa-miR-196a-5p on cell proliferation and apoptosis.** (**A**) The effect of *LOC134466* on hsa-miR-196a-5p expression was determined by dual-luciferase reporter gene assay. (**B**) The effects of hsa-miR-196a-5p on TAC1 expression was determined by dual-luciferase reporter gene assay. (**C-D**) The effects of hsa-miR-196a-5p on cell proliferation were assessed by colony formation assay. (**E-F**) The effects of hsa-miR-196a-5p on cell apoptosis was assessed by Annexin V/PI double staining and apoptosis rates were calculated. (**G**) The effect of LOC134466/hsa-mir-196a-5p/TAC1 axis on EC was analyzed by plate clone formation assay. (**H-J**) The roles of *LOC134466*/hsa-miR-196a-5p/*TAC1* axis on cell apoptosis and cell proliferation were determined by Annexin V/PI double staining and colony formation assay respectively. *P<0.05, **P<0.01, compared with vector group. #*P*<0.05, compared with *LOC134466* group, &*P*<0.05 compared with *TAC1* group, $$*P*<0.01 compared with hsa-miR-196a-5p group.

Dual-luciferase reporter gene assay demonstrated that hsa-miR-196a-5p was significantly down-regulated upon *LOC134466* overexpression (Figure 10A) and *TAC1* was also remarkably decreased through hsa-miR-196a-5p treatment (Figure 10B). Figure 10A-B indicated that *LOC134466* promoted TAC1 expression by downregulating hsa-miR-196a-5p. Then, we analyzed the impact of hsa-miR-196a-5p on cells proliferation, apoptosis in KLE cells. It was found that hsa-miR-196a-5p promoted cell proliferation (Figure 10C-10E) and inhibited apoptosis (Figure 10C&10F). Besides, the effect of *LOC134466*/hsa-mir-196a-5p/*TAC1* axis on EC was analyzed by a series of cell experiments. From the perspective of cell proliferation, cell proliferation was significantly reduced in both LOC134466 and TAC1 treatment group, but a totally opposite effect was produced by hsa-miR-196a-5p mimics treatment (Figure 10G-10I). Cell apoptosis, on the other hand, was remarkably decreased with LOC134466 and TAC1 treatments, but increased by hsa-miR-196a-5p inhibit treatment (Figure 10G&10J). To conclude, *LOC134466*/hsa-miR-196a-5p/TAC1 axis played important roles in the pathogenesis of EC.

### *LOC134466*/hsa-miR-196a-5p/*TAC1* axis affected EC carcinogenesis through activating neuroactive ligand-receptor interaction

**Figure 11:**
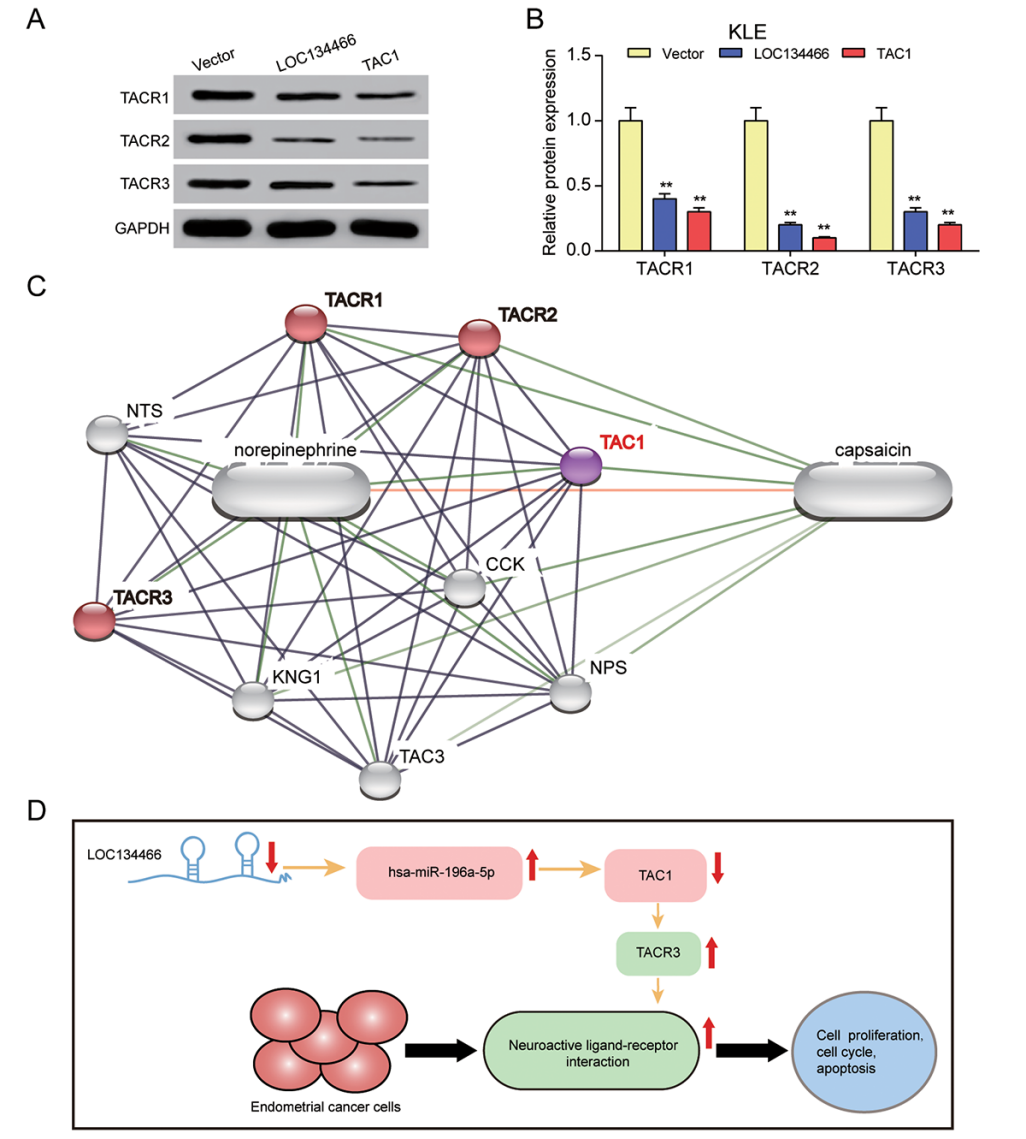
***LOC134466*/hsa-miR-196a-5p/*TAC1* axis affected EC carcinogenesis through neuroactive ligand-receptor interaction.** (**A-B**) The effects of *LOC134466* or *TAC1* overexpression on TACR1, TACR2 and TACR3 expression were determined by western blot. Protein expression was normalized to that of GADPH. (**C**) The *TAC1* network was generated by Stich website. The neuroactive ligand-receptor interaction was marked red. (**D**) A model of *LOC134466*/hsa-miR-196a-5p/*TAC1* axis action on EC development. ** *P*<0.01 compared with corresponding control group.

Compared with the vector control, enhanced expression of *LOC134466* and *TAC1* caused a sharp decrease of several known neuroactive ligand-receptor interaction targets, including TACR1, TACR2 and TACR3 in KLE cells (Figure 11A-11B). Figure 11C illustrated that *TCA1* directly associate with TACR1, TACR2 and TACR3. As mentioned previously, TAR3 was overexpressed in EC tissues (Figure 5A). So, we hypothesized that *LOC134466*/hsa-miR-196a-5p/*TAC1* axis performed tumor suppressive function through activating neuroactive ligand-receptor interaction (Figure 10D).

### *LOC134466* and *TAC1* suppressed tumor growth *in vivo*

**Figure 12:**
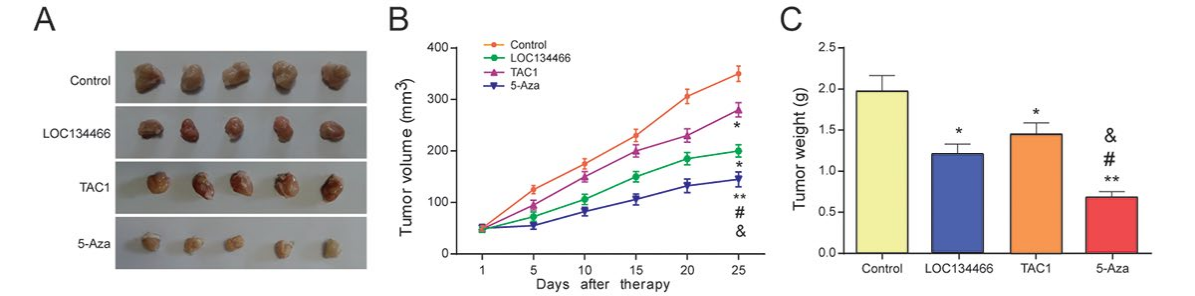
***LOC134466* and *TAC1* suppressed tumor growth *in vivo*.** (**A-C**) Tumor growth was assessed by tumor volume and tumor weight. Five-week nude mice were injected with KLE cells (1× 10^6^/200 μl PBS) subcutaneously. * P<0.05, ** *P*<0.01, compared with control group, #*P*<0.05, compared with *LOC134466* group, &*P*<0.05 compared with *TAC1* group.

To verify the curative effect of different treatments in EC, Tumor Xenograft experiment was employed. The results of Xenograft assay showed in Figure 12A-12C revealed that the tumor size and weight were suppressed in *LOC134466* group, *TAC1* group and 5-Aza treatment group.

## DISCUSSION

The results of bioinformatic analysis and *in vitro* studies together revealed a novel regulatory axis, *LOC134466*/hsa-miR-196a-5p/*TAC1* axis that play an important tole in EC development. Moreover,we found that both *LOC134466* and its target gene *TAC1* was hypermethylated in EC tumor tissues. Upregulating *LOC134466* and *TAC1*, and 5-Aza treatment sucessfully arrested tumour progression *in both* in vitro and *in vivo* studies. All together, *LOC134466*/hsa-miR-196a-5p/*TAC1* axis may be an important therapeutic targets for EC.

*LOC134466* is also known as *ZNF300P1*, a pseudogene of the human zinc finger protein ZNF300 [23]. Notably, *LOC134466* has been recently proved to be a novel methylated marker [24] and many researches have shown that *LOC134466* is commonly silenced by methylation in several cancer types such as epithelial ovarian cancer and small cell lung cancer [23,24]. These research results potentially indicated that *LOC134466* methylation may be a major factor in carcinogenesis. On the other hand, *TAC1* is a single-copy gene with 7 exons [25], which encodes neuroendocrine gastrointestinal peptides including substance P (SP) and neurokinin A [26]. Both substance P (SP) and neurokinin A play vital roles in cancer development [25,27,28]. In order to verify the role of *LOC134466* and *TAC1* in tumorigenesis of EC, we constructed *LOC134466* and *TAC1* overexpressed KLE cell line models through DNA vector. The results showed that *LOC134466* and *TAC1* inhibited EC cells proliferation and accelerated apoptosis. Via methylation-specific PCR (MSP), it was found that both *LOC134466* and *TAC1* were hypermethylated in the EC tissues and hypermethylation resulted in their low expression. Development of endometrial carcinoma was significantly suprresed by 5-Aza treatment. To conclude, both LOC134466 and TCA1 played important roles in EC development.

Many studies over the past ten years have begun to reveal the interaction between lncRNA-miRNA-mRNA [14,16,29]. However, little research has reported the structural and functional relationship between *LOC134466* and *TCA1*. Thus, it’s very essential to figure out whether *LOC134466* and *TCA1* are functionally related. In this research, we reported how *LOC134466* and *TAC1* interact for the first time. The results showed that hsa-miR-196a-5p was a bridge between *LOC134466* and *TAC1*. Based on the further analysis, we finally confirmed that the *LOC134466* / hsa-miR-196a-5p / *TAC1* axis regulated endometrial carcinoma by *TACR3*. It should be emphasized that our research has revealed that *TACR3* which was recently found to be associated with gingival oral squamous cell carcinoma [30,31] also played significant roles in EC. Therefore, we can reasonably speculate that *TACR3* is a key impact factor of many cancers.

TACR3 can activate neuroactive ligand-receptor interaction. Recently, some researches have shown that neuroactive ligand-receptor interaction, a kind of G protein-coupled receptor-mediated signaling pathway [32], is associated with cancer progression such as bladder cancer and pancreatic cancer [32–34]. As the first gate, the high activity of GPCRs may lead to transportation of externally unfavorable signals such as glucose, insulin, or carcinogens into a cell which may induce a series of cascade reactions related to carcinogenesis [32]. Given the heatmap and GSEA report, we found that in EC, the neuroactive ligand-receptor interaction was also significantly up-regulated. According to these results, apparently, neuroactive ligand-receptor interaction plays an important role in EC carcinogenesis.

In conclusion, our research has revealed that *LOC134466* methylation was directly related to tumorigenesis of EC, but it is worthy of further study on the relationship between *LOC134466* methylation and other cancers. Furthermore, we also identified its internal mechanism that could be summarized as a novel regulatory axis, *LOC134466* / hsa-miR-196a-5p / *TAC1*, which activated neuroactive ligand-receptor interaction through activating TACR3 in EC. However, whether the LOC134466 / hsa-miR-196a-5p / TAC1 axis we built exists in other kinds of cancers and how it plays roles respectively need to be further explored.

## MATERIALS AND METHODS

### Bioinformatic analysis

As a kind of functional pipeline, the Chip Analysis Methylation Pipeline (ChAMP) package not only combines the available 450k analysis methods at present, but also provides its own original functionality. On the other hand, Gene set enrichment analysis (GSEA) is used to judge whether a set of genes that are priori defined exhibits statistically significant, consistent differences between two biological states. In this study, we detected gene methylation by R through ChAMP package. Then, an ordered list of all genes was generated by GSEA according to their correlation with endometrial carcinoma. The significant difference was observed towards high- and low-activity related pathways between endometrial cancer group and normal group.

### Tissue samples and cell culture

20 endometrial tissue samples were obtained from endometrial carcinoma patients who presented at Huangjiahu Hospital of Hubei University of Chinese Medicine. The study was performed in accordance with human subject guidelines and was approved by the Scientific and Ethical Committee of Huangjiahu Hospital of Hubei University of Chinese Medicine. All subjects gave written informed consent.

The cell lines of endometrial cancer, KLE, Ishikawa, HEC1A, and the normal endometrial cell line hEEC were originally from BeNa Culture Collection (Beijing, China). Cultured the KLE, Ishikawa, and hEEC cells in 90% EMEM (AmyJet Scientific, Wuhan, China), which was replenished with 10% fetal bovine serum (FBS; Thermo Fisher Scientific, Waltham, MA, USA). At the same time, cultured the HEC1A cells in 90% McCoy’s 5a medium (Thermo Fisher Scientific) replenished with 10% FBS. All cells were grown at 37 °C, 5% CO_2_.

### Methylation-Specific PCR (MSP)

It has been previously described and shown in Table 1 about what the methylation and unmethylation-sensitive primers of *LOC134466* and *TAC1* were used in the study. In a 25 μl reaction mixture, we amplify 1.5 μl of bisulfite-converted DNA. The reaction mixture contained 200 μM dNTPs, 10X reaction buffer, 2.5 mM MgCL_2_, 10 pM forward and reverse primers, as well as 1 U of FastTaq (Roche, Basel, Switzerland). Bisulfite-modified DNA was then amplified with two primer sets that differentiate methylated from unmethylated DNA. Meanwhile, we performed Hot-start PCR at an annealing temperature of 60 °C using hot-start Taq DNA polymerase (Thermo Fisher Scientific). Cases in which methylated alleles were present were repeated once again for confirmation.

**Table 1:** Sequences of MSP primers.

**Name**	**Sequence**
Methylation	
*LOC134466* forward	5' TACGAGGTTAGGAGTTC 3'
*LOC134466* reverse	5' AATAACACGATCTCGA 3'
*TAC1* forward	5' GGGGCGTTAGATTTGTAGAC 3'
*TAC1* reverse	5' ACGATAACTCGTCGATACCC3'
No Methylation	
*LOC134466* forward	5' TACGAGGTTAGGAGTTC 3'
*LOC134466* reverse	5' AATAACACGATCTCGA 3'
*TAC1* forward	5' GAGGGGGTGTTAGATTTGTAGAT 3'
*TAC1* reverse	5' AAAACAATAACTCATCAATACCC 3'

### The isolation of RNA and quantitative real time PCR

We used TRIzol reagent (Invitrogen, Carlsbad, USA) to extract the total RNA endometrial carcinoma tissues and corresponding healthy tissues. The SuperScriptTM II Reverse Transcriptase Kit (Invitrogen) was used to reversely transcript of 5 μg of total RNA. SYBRGreen reagent (Thermo Fisher Scientific) was used for quantitative real time polymerase chain reaction (qRT-PCR). For qRT-PCR, primers that were used to estimate the expression of total *LOC134466* and *TAC1* were listed in Table 2. The expression of these genes was normalized to that of *GADPH*. Besides, subsequent to the real-time RT-PCR, we examined the dissociation reaction diagram and then verified the specificity of the PCR. It is worth conducting all the experiments in triplicate.

**Table 2:** Sequences of primers for qPCR.

**Name**	**Sequence**
*LOC134466* Forward	5’-AGTACGGGGTCGTTATTTTGAGATTTT-3’
*LOC134466* Reverse	5’-CGGAATGTGGATCCCTTCICAATCACTATAATACAA-3’
*TAC1* Forward	5’-TGGTCCGACTGGTACGACAG-3’
*TAC1* Reverse	5’-CTGCAGAAGATGCTCAAAGGG-3’

### 5-Aza treatment studies

Use MTT assay to evaluate the cytotoxicity of 5-Aza-2-Deoxycytidine. Concisely, each well of the 96-well plate contained 50 μL of RPMI medium was seeded with 5×104 cells (KLE, Ishikawa, HEC1A). 24 h later, added 5-Aza-2-Deoxycytidine of various concentrations (0, 2.5, 5, 7.5, 10, 12.5 and 15 μM) and 50 μL of MTT (5 mg/mL stock solution)into each well and after 48 h. Incubated the plates for an additional 4 h. Finally, discard the medium and dissolve the substance formed in the cells by 50 μL DMSO. At last, measure the optical density at 570 nm by Multiskan™ Sky microplate spectrophotometer (Thermo Fisher Scientific). To evaluate the effects of 5-Aza on gene expression, endometrial cancer cells were seeded on 10 cm plate and after 24 hours treated with 2 μmol/L of the DNA demethylating agent 5-Aza-2’-deoxycytidine (Sigma-Aldrich, St. Louis, MO, USA) for 72 h. Gene expression was assessed by qRT-PCR.

### Cell transfection

To increase *LOC134466* and *TAC1* expression, *LOC134466* expression vector and TAC1 expression vector were purchased from Biovector (Beijing China). For hsa-miR-196a-5p treatment, use Lipofectamine 3000 reagent (Thermo Fisher Scientific) to transfect cells separately with hsa-miR-196a-5p mimics in the light of the manufacturer’s protocol.

### Colony formation assay

Seeded the transfected cells in 96-well plates at the density of 5 × 10^3^ cells /well, and incubated the plates for 48 hours. After incubation, the resultant colonies were stained by 0.2% crystal violet and those larger than 1 mm in diameter were counted under the microscope. The experiments were repeated in triplicate and cell density was indirectly assessed by the absorbance at 570 nm.

### Cell cycle assay

For cell cycle analysis, 2×10^5^ cells were plated in a 6-well culture plate and grown for 24 h. Cells were then incubated with 1 mM thymidine (Sigma-Aldrich) for 24 h to synchronize cells at the G1/S boundary. The cells were then treated with serum-deprived culture medium for another 24 h. Next, the cells were trypsinized, then washed twice using cold PBS and fixed with cold 70% ethanol at -20°C overnight. After an overnight stay, washed the cells twice with PBS and incubated them with 10 mg/ml RNase A and 400 mg/ml propidium iodide (PI; MedChemExpress, Shanghai, China) in PBS at room temperature for 30 mins.

### Flow cytometry

5×10^5^ cells of each well were cultured in 12-well plates for 48 hours at 37 °C in a 5% CO_2_ atmosphere. And after fixed with 70% ethanol for 72 h and stained by 25 μg/mL PI in fluorescence-activated cell sorting buffer (PBS containing 0.1% of bovine serum albumin, 0.05% of Triton X-100, and 50 μg /mL RNaseA), incubated cells for 30 min in dark environment at room temperature. Then used a Attune NxT Flow Cytometer (Thermo Fisher Scientific) to assess relative cell numbers in each cell-cycle phase (G1, S, G2, and subG1). Each experiment was performed in triplicate.

### Dual-luciferase reporter gene assay

KLE cells were used to perform the dual-luciferase reporter assay. For hsa-miR-196a-5p reporter assay, cells were co-transfected with 250 ng of *LOC134466* expression vector or control. For TAC1 reporter assay, cells were co-transfected with 250 ng of hsa-miR-196a-5p expression vector or control. According to the manufacturer’s protocol, we used the Dual-Luciferase Reporter Assay System (Biomart, Beijing, China) to measure luciferase activity. For each transfection, three replicates were performed to get the activity of luciferase.

### Western blot

Washed cells twice with PBS (Thermo Fisher Scientific), and then collected and lysed them with RIPA buffer (Beyotime, Shanghai, China). The protein concentrations in the cell lysates were measured using a BCA Protein Assay Kit (Thermo Fisher Scientific). Separated the protein samples in 12% SDS-PAGE gel, and then samples were transferred onto the PVDF membranes (Thermo Fisher Scientific). Blocked the membranes with 5% nonfat dry milk and probed them with primary antibodies (anti-ZNF300P1, Sigma; anti-TAC1, Thermo Fisher Scientific). After being incubated overnight at 4 °C, the membranes were washed and incubated with a secondary peroxidase conjugated antibody for 1 h. Antibody binding was detected using Amersham ECL detection reagents (GE Healthcare, Little Chalfont, Buckinghamshire, UK) via X films. All blots were stripped and reprobed with polyclonal anti-GADPH antibody to ascertain equal loading of proteins. Repeated each experiment for three times.

### Tumor xenograft

Five-week nude mice at the same condition were arranged randomly, with five in each group, including control group, *LOC134466* group, TAC1 group and 5-Aza group. Then injected KLE cells (1× 10^6^/200 μl PBS) subcutaneously into the nude mice at a single site. Used calipers to measure the size of tumor weekly. When the average tumor size reached about 100 mm^3^, injected 5-Aza-dC (5 mg/kg) and measured the tumor volumes and tumor weights every 5 days. 25 days after simultaneous administration, all mice were sacrificed and whole proteins were isolated from the xenografted tissues of the mice for western blot analysis and immunohistochemical analysis. The Animal Care and Use Committee of Huangjiahu Hospital of Hubei University of Chinese Medicine approved all these experiments. And we obeyed the institutional ethics guidelines when we done the animal experiments.

### Statistical analysis

Use GraphPad Prism 6.0 to conduct statistical analyses. Present all the values as means ± SD and we used the Bonferroni correction method to correct the multiple testing in order to control the false discovery rate (FDR). Student t-test was performed to compared between two groups and One-way ANOVA was used for comparison among multiple groups. The level of significance was assigned at p-value < 0.05.

### Declarations

**Ethics approval and consent to participate:**This study was authorized by Huangjiahu Hospital of Hubei University of Chinese Medicine, and obtained written informed consents from all the participants.

**Availability of data and material:** All data generated or analyzed during this study are included in this published article.

## SUPPLEMENTARY MATERIAL

Supplementary Figure S1
