# Supplementary material for: LOC134466 methylation promotes oncogenesis of endometrial carcinoma through LOC134466/hsa-miR-196a-5p/TAC1 axis
Source: Aging (Albany NY). 2018 Nov 28;10(11):3353–70. doi: 10.18632/aging.101644 (PMC6286819; doi:10.18632/aging.101644)
Supplement: Supplementary Figure S1 [file aging-10-101644-s001.pdf]

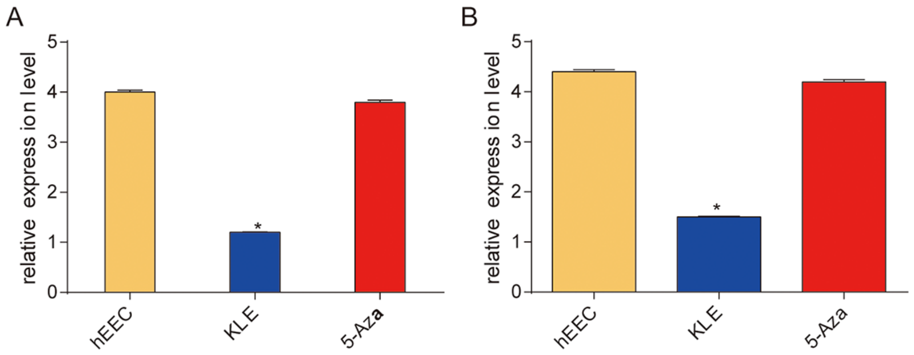

Figure S1. 5-Aza treatment upregulated *LOC13446* and *HAC1* expression in EC. (**A**): The relative expression of *LOC134466* in each group. (**B**) The relative expression of *TAC1* in each group. The gene expression was determined by qRT-PCR. *$P<0.05$.
